# Seasonal dynamics of the juvenile fish community structure in the Maowei Sea mangroves

**DOI:** 10.1371/journal.pone.0192426

**Published:** 2018-02-13

**Authors:** Zhi-Qiang Wu, Qi Zou, Tao Chang, Dong Zhang, Liang-Liang Huang

**Affiliations:** 1 College of Environmental Science and Engineering, Guilin University of Technology, Guilin, Guangxi Province, China; 2 Guangxi University, Nanning, Guangxi Province, China; 3 Qinzhou Marine Administration, Qinzhou, Guangxi Province, China; Tanzania Fisheries Research Institute, UNITED REPUBLIC OF TANZANIA

## Abstract

More than 50% of Chinese mangroves were lost between 1950 and 2000 to habitat destruction, prompting an urge for conservation. To assess the importance of the protected Maowei Gulf mangrove estuary for fish population assemblage in the Beibu Gulf (China), we studied species composition and abundance of juvenile fish (including larvae) from July 2012 to June 2013. A total of 11 691 specimens were collected, which belonged to 24 species and 15 families. Six perciform species constituted 93% of the total sample. *Pseudogobius javanicus* (53.29%) was the dominant species from August to November, *Omobranchus elegans* (28.49%) from April to July, non-identified species in December and January, and *Liza carinata* in February and March. A number of commercially important fish species were also identified. Abundance was the highest in summer/early autumn (max 162.4 in Sep), and lowest in winter/early spring (Mar = 4.5). Diversity (H’) and richness (D_ma_) indices (both max. in May: 1.67 and 1.95 respectively) were generally positively correlated with tide and temperature, and negatively with salinity. Seasonal variations play a more important role in the fish assemblage structure than tidal rhythm, with differences particularly pronounced between colder and warmer months. Despite the prominent seasonal differences in abiotic factors, this study indicates that Maowei mangroves provide habitat and food for juvenile fish throughout the year and thus are indispensable for the fish diversity in the Beibu Gulf.

## Introduction

The importance of mangroves, forested wetlands found along tropical and subtropical shorelines around the globe, is widely accepted [1–4]. High fish species diversity (often more than 100) in mangrove estuaries highlights the central role that mangroves play in the biology and ecology of fishes in (sub)tropical inshore and estuarine areas [5]. Mangroves are characterized by high structural complexity and high supply of food, which makes them a very suitable habitat for juvenile fish [6]. In comparison to other habitats, fish assemblages are unique in mangroves, which indicates their importance not only as nursery sites but also for the overall fish species diversity [7–10]. Rapid global loss of mangrove forests has prompted the need for conservation [2,5], particularly in China, where more than 50% of mangroves were lost between 1950 and 2000 as a result of habitat destruction [11]. Although the protection is based mostly on their importance for fisheries and endangered fish species, the actual importance of mangrove habitats for commercial fisheries is still debated [5,12,13]. Thus, in the interest of both sustainable fisheries and mangrove management, it is necessary to better understand the interconnectedness of mangroves and fishes [5].

Since the early 1990s Chinese government has begun to make efforts towards the establishment of mangrove natural reserves and mangrove reforestation [11]. An early part of those efforts was the establishment of The Maowei Sea National Marine Park at the far northern end of the Gulf of Tonkin (known as Beibu Gulf in China), with a mangrove area of approximately 30 km^2^ [14]. However, this only took place after approximately 70% of mangrove forests in the Beibu Gulf were damaged or lost [15]. Although several studies have been conducted on this locality, e.g. [14,16,17], the importance of this mangrove wetland as a fish nursery and its role in the protection of fishery resources is still not fully understood. Furthermore, all those studies were published in local journals in Chinese language. This reflects the general national trend, where approximately 75% of studies about Chinese mangroves have been published in Chinese language [11], thus limiting the amount of data available to the wider scientific community. Furthermore, the Southeast (SE) Asian region is generally still underrepresented in the published literature about mangroves and fishes [12]. This scarcity of data hampers the progress in the understanding of the role that mangroves play in the maintenance of fish community stability and diversity, especially in the light of intensive commercial fisheries in the region. Therefore, we investigated seasonal variations of juvenile fish communities in the Maowei Sea mangroves during the period of one calendar year. The focus was on juvenile fish (which included larvae), as they remain understudied in comparison to adult fish assemblages in mangroves [18]. As the full understanding of interrelationships between fishes and mangroves also requires a good understanding of a number of abiotic factors [5,19], we have also studied the impacts of temperature, salinity and tidal rhythm on the fish community structure. Finally, we discuss these results in the light of the overfishing and dramatic fish resource depletion in the Beibu Gulf [20–22]. This study provides a scientific base [13] for the effective protection of fish diversity in the Beibu Gulf, and therefore data crucial for the marine resource management and conservation in China [15].

## Results

### Sampling and hydrographic conditions

**Fig 1:**
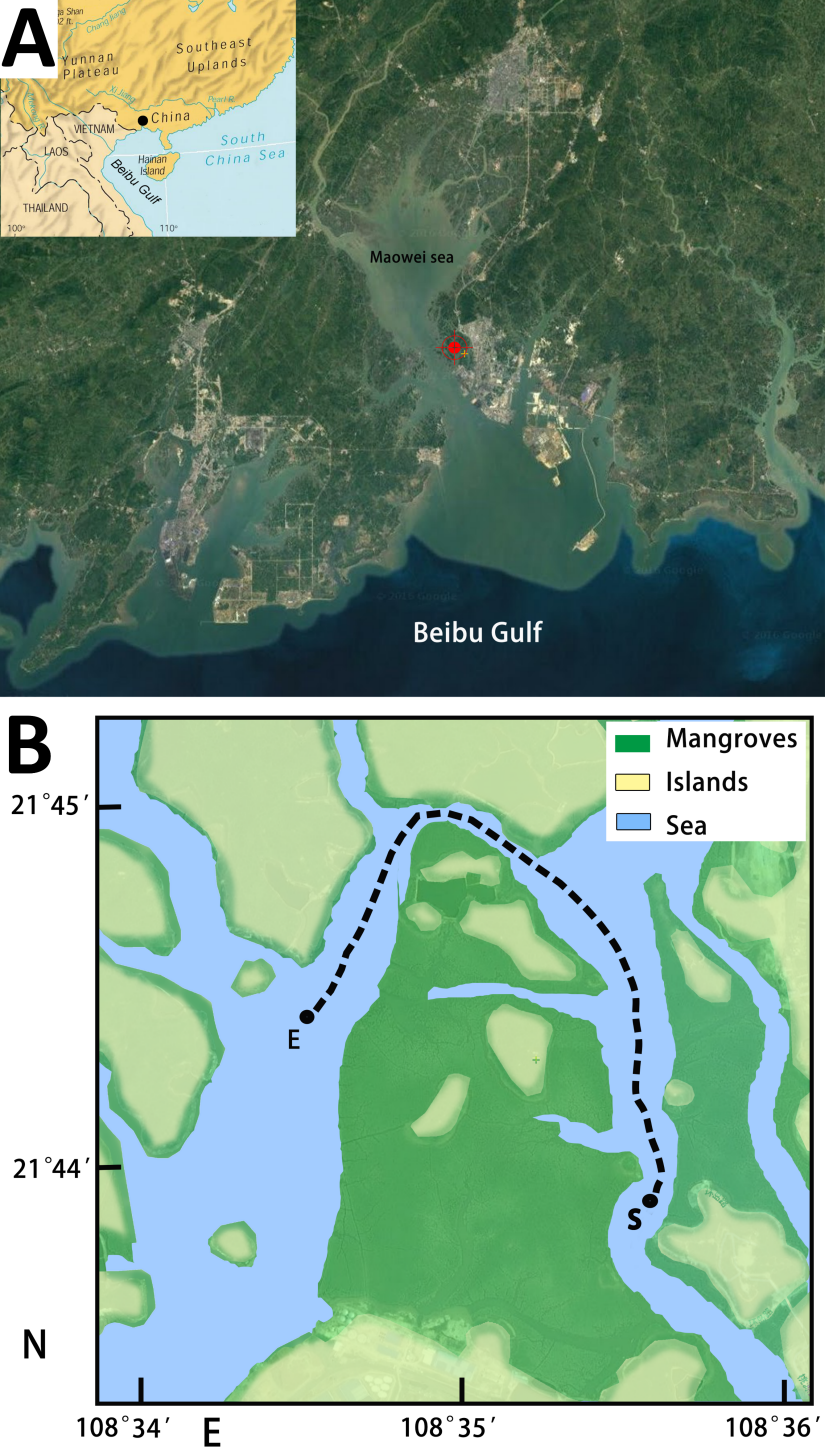
**The sampling location (a) and path (b) in the Maowei Sea mangroves.** a) Sampling location is marked by a black dot (small picture) and a red dot (large picture). b) S—starting point, E—ending point.

**Fig 2:**
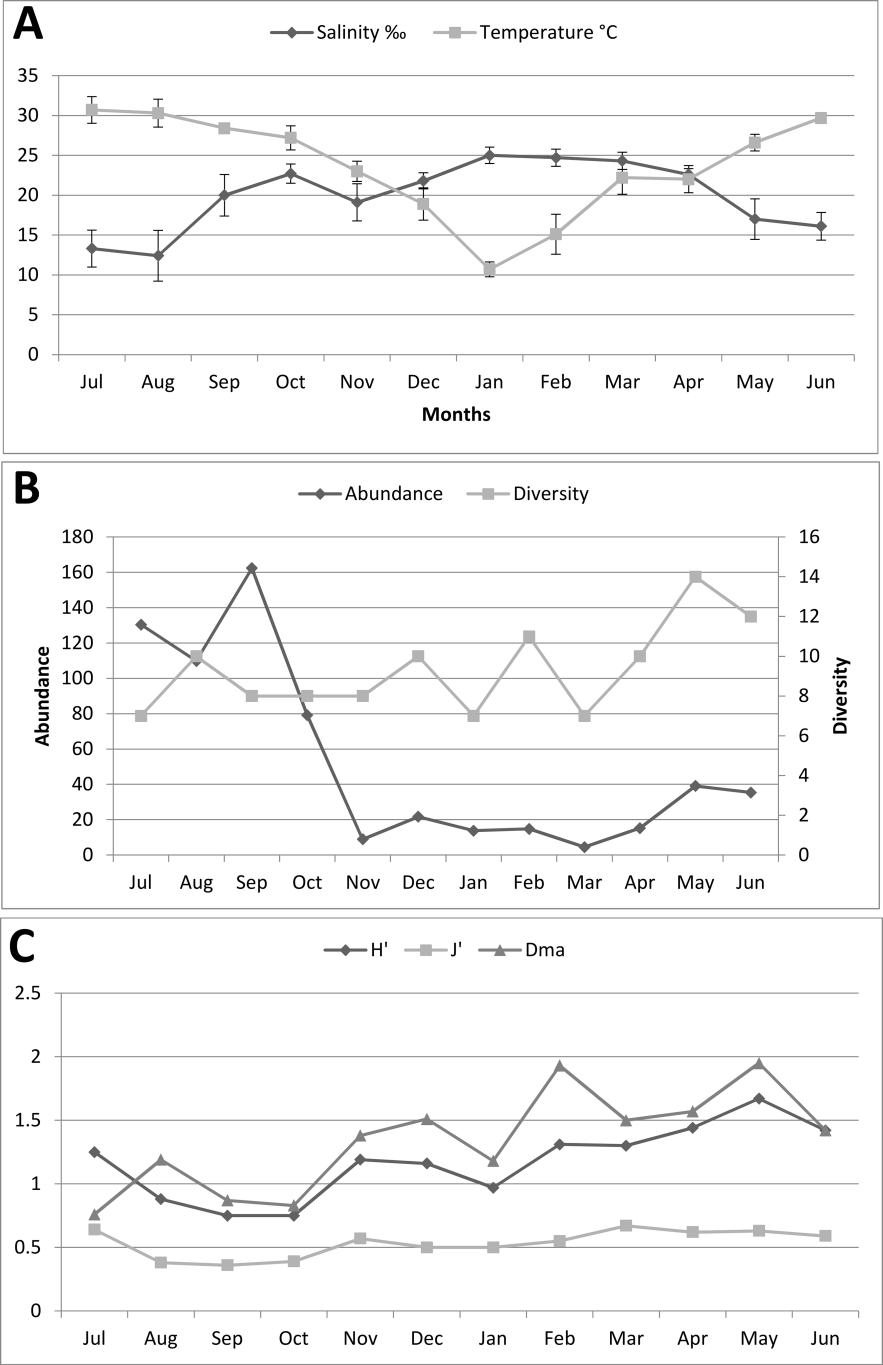
Monthly variation in abiotic factors, species diversity index and abundance in the Maowei Sea mangroves. a) Monthly variation in temperature and salinity. b) Monthly variation in diversity and abundance, where diversity is the total number of sampled species and abundance is the total number of sampled specimens. c) Monthly variation in diversity indices in the studied area, where H’ is Shannon-Weaver's index of species diversity, D_ma_ is Margalef’s index of species richness, and J’ is Pielou's index of evenness.

Fish juveniles (includes larvae) were sampled among mangrove islands within the channel that separates the Maowei Sea from the Beibu Gulf (Fig 1) from July 2012 through June 2013. Average monthly water temperature in the study area (Fig 2A) ranged between 10.7°C in January and 30.7°C in July (average annual temp. = 23.7°C). Maowei Sea is an estuary separated from the Beibu Gulf by a narrow channel (Fig 1A), so salinity is under a strong influence of the rivers disemboguing into the estuary [23]. As a result of this, a very prominent variability in the average monthly salinity, ranging from 12.4‰ in August to 25.0‰ in January (annual average = 19.9‰), was observed during the study period (Fig 2A). Generally, salinity was relatively low during the rainy season, between May and August (≤ 17‰), and relatively high during the dry season, from September to April (> 19‰).

### Fish fauna

A total of 11 691 fish juveniles were collected and pooled, comprising 24 species and 15 families (Table 1). Fish juveniles (larvae) that were in unidentifiable early life stages were classified as “other”. Perciformes were the most abundant order, comprising 7 families, 14 species, and more than 91.15% of the total sampled specimens. *Pseudogobius javanicus* (Perciformes, Gobiidae) was the most dominant species, constituting 53.29% of the total catch. It was followed by *Omobranchus elegans* (Perciformes, Blenniidae) with 28.49%, *Nemipterus virgatus* (6.30%), “other” (2.61%), *Konosirus punctatus* (2.24%), and *Engraulis japonicus* (1.44%). These six species constituted approximately 95% of the total catch.

**Table 1:** Fish species sampled between July 2012 and June 2013 in the Maowei Sea mangroves.

Order	Family	Species	Month	No	%
Perciformes	Gobiidae	*Pseudogobius javanicus*	7–12, 2–6	6230	53.29
		*Luciogobius sp*.	8, 12, 2–4, 6	10	0.09
		*Caragobius urolepis*	5	21	0.18
		sp. 1	2–4	46	0.39
		sp. 2	2–3, 5–6	30	0.26
	Blenniidae	*Omobranchus elegans*	7–11, 4–6	3331	28.49
	Nemipteridae	*Nemipterus virgatus*	7–11, 4–6	736	6.30
	Ambassidae	spp.	8–10, 4–6	116	0.99
	Eleotridae	*Ophiocara* spp.	8–12, 5	32	0.27
		*O*. *porocephala*	12, 1–2	44	0.38
	Callionymidae	*Repomucenus olidus*	8–12, 1–2	16	0.14
	Sparidae	*Acanthopagrus latus*	12, 1	16	0.14
		*A*. *schlegeli*	12, 1–3	22	0.19
		*Rhabdosargus sarba*	5	6	0.05
Clupeiformes	Clupeidae	*Konosirus punctatus*	7–11, 4–6	262	2.24
		*Sardinella* spp.	1–4	26	0.22
	Engraulidae	*Engraulis japonicus*	7–8, 5–6	168	1.44
Syngnathiformes	Syngnathidae	*Parasyngnathus argyrostictus*	7–11, 4–6	79	0.68
Gadiformes	Bregmacerotidae	*Bregmaceros* spp.	11–12	36	0.31
Pleuronectiformes	Cynoglossidae	spp.	12, 5	2	0.02
Tetraodontiformes	Tetraodontidae	spp.	1–3	7	0.06
Mugiliformes	Mugilidae	*Liza carinata*	2–5	140	1.20
Aulopiformes	Synodontidae	spp.	5–6	10	0.09
N.A.	N.A.	“other”	12, 1–2	305	2.61

Higher taxonomic levels (order and family) are given in the two columns on the left. Months are in the numeric form (1 to 12). “No” is the total number of specimens and % is the numeric percentage of each species in the total sample.

However, dominant species varied between seasons (Table 2). *Pseudogobius javanicus* was dominant from Aug to Nov (max. in Sep at 2280 individuals). It was present every month except January, but its numbers remained very low in Feb (2) and Mar (6). *Omobranchus elegans* was abundant (133 to 1 263 individuals) from Apr to Oct, dominant from Apr to Jul (max. abundance), and completely absent from Dec to Mar. Unknown “Other” species was (or were) dominant in Dec (189) and Jan (115), and completely absent during the remaining months, apart from a single specimen recorded in Feb. *Liza carinata* was present only in spring (Feb to May), and dominant in Feb and Mar.

**Table 2:** Dominant fish species in the Maowei Sea mangroves between July 2012 and June 2013.

Dominant species	Jul	Aug	Sep	Oct	Nov	Dec	Jan	Feb	Mar	Apr	May	Jun	Sum
***Pseudogobius javanicus***	821	1379	2280	1108	103	151	0	2	6	93	226	61	6230
***Omobranchus elegans***	1263	468	669	206	28	0	0	0	0	133	281	283	3331
***Gobiidae* spp 1**								31	12	3			46
***Liza carinata***								66	24	7	43		140
**Other**						179	125	1					305
**Total abundance**	2477	2196	3085	1343	163	391	166	168	54	304	782	562	11691

Fish juveniles that were unidentifiable (to a family-level) in the early life stages were classified as “other”. Total abundance is the number of all specimens sampled each month.

The average monthly fish abundance was highest from Jul to Sep 2012. It peaked in Sep at 162.4 individuals per single tow. This was followed by a dramatic decrease in abundance through Oct (79.1) and Nov (8.9). The abundance then remained very low (min. 4.5 in Mar, max. 21.7 in Dec) until April. An uptick was observed in May and June 2013, but the numbers (35–40 individuals per tow) were much lower than in July 2012 (130.4 individuals per tow). Fish species diversity (the total number of sampled species per month), however, was relatively stable throughout the year (mostly between 7 and 11 species), with an uptick observed in May (14 species) and June (12 species) 2013 (Fig 2B). Margalef’s index of species richness (D_ma_) and Shannon-Weaver's index of species diversity (H’) exhibited very similar temporal patterns (correlation between the two trends was 0.75): both exhibited an increasing trend with similar monthly oscillations (Fig 2C). However, a few notable differences were observed: D_ma_ values were higher than H’ values, except in July 2012, when D_ma_ was at its lowest point (0.76), whereas H’ (Jul = 1.25) would only reach its lowest point in Sep and Aug (0.75). The index of species evenness (J’) remained rather stable throughout the year, with lowest values observed from August to October.

### Fish assemblage structure in relation to seasonal and tidal changes

**Fig 3:**
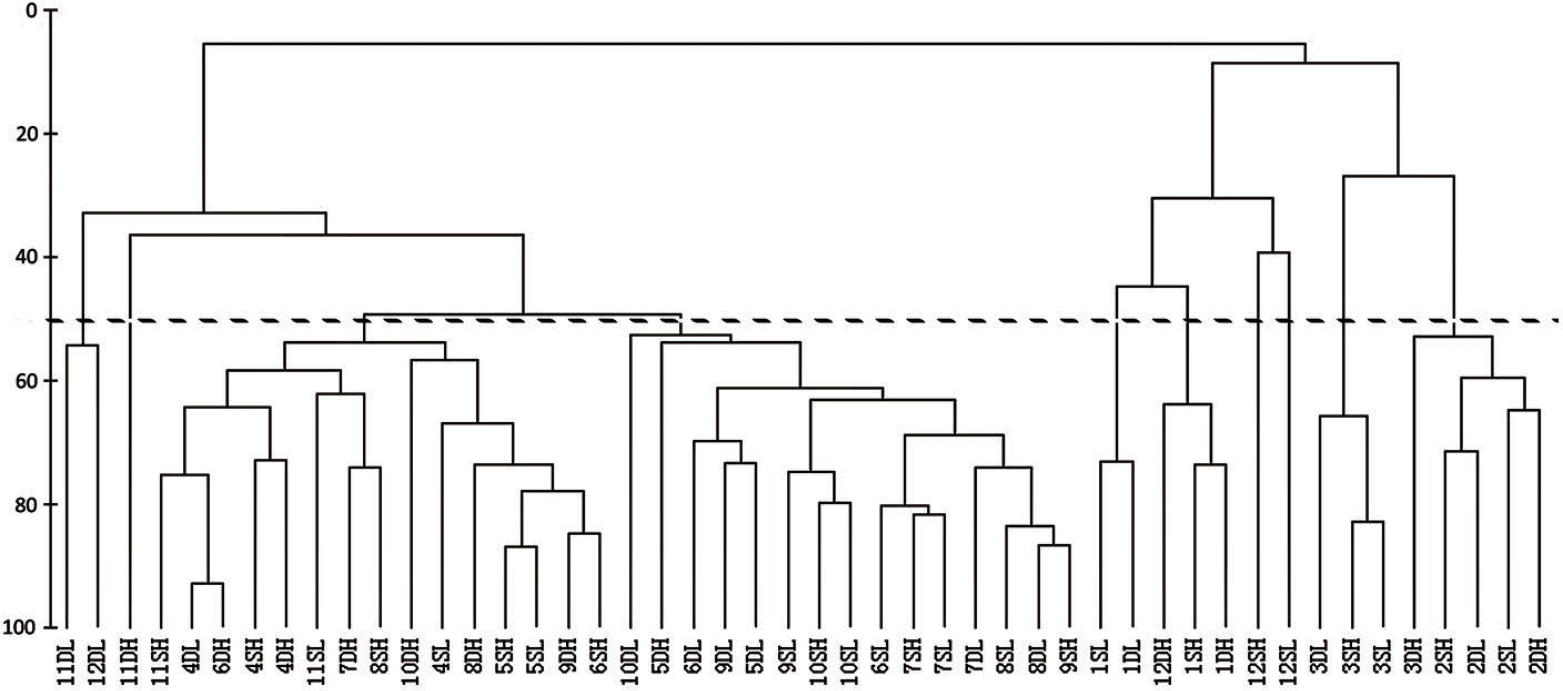
Cluster analysis of the fish assemblage structure by monthly variations and tidal rhythm. Monthly samples were divided according to the tidal rhythm into the following four categories: high tide during semi-diurnal tides (SH), low tide during semi-diurnal tides (SL), high tide during diurnal tides (DH) and low tide during diurnal tides (DL). Months are in the numeric form (1 to 12). Dashed line indicates the 50% cut-off value.

According to the tidal rhythm, we have divided monthly samples into the following four categories: high tide during semi-diurnal tides (SH), low tide during semi-diurnal tides (SL), high tide during diurnal tides (DH) and low tide during diurnal tides (DL). Thus classified, the total number of samples (206) yielded 48 samples across 12 months, which were subsequently submitted to a cluster analysis using Bray-Curtis similarity to infer temporal variations within the juvenile fish assemblage in the study area (Fig 3). Two distinct clusters were observed, one (I) containing all samples from Dec to Mar, apart from one Dec sample (12DL), and the other (II) containing the samples from the remaining eight months. Cluster I was further divided into two sister sub-clusters: one containing Dec and Jan samples, and the other containing Feb and Mar samples. In cluster II, Nov and Dec DL samples formed a separate sub-cluster. The November DH sample was placed in a separate branch, whereas the remaining samples were evenly (15 in each) divided into two sister clusters, but without a discernible pattern. Clustering according to the tidal rhythm was minimal, which indicates that seasonal variations play a more important role in the fish assemblage structure than tidal rhythm. These large differences in fish assemblage between colder (Dec, Jan, Feb and Mar) and warmer months were further corroborated by the ANOSIM test (Table 3).

**Table 3:** One-way ANOSIM test for juvenile fish communities between different months by R(P).

	Feb	Mar	Apr	May	Jun	Jul	Aug	Sep	Oct	Nov	Dec
Jan	0.94 **(2.9)**	1.00 **(2.9)**	1.00 **(2.9)**	1.00 **(2.9)**	1.00 **(2.9)**	1.00 **(2.9)**	1.00 **(2.9)**	1.00 **(2.9)**	1.00 **(2.9)**	1.00 **(2.9)**	0.31 (14.3)
Feb		0.83 **(2.9)**	1.00 **(2.9)**	1.00 **(2.9)**	1.00 **(2.9)**	1.00 **(2.9)**	1.00 **(2.9)**	1.00 **(2.9)**	1.00 **(2.9)**	1.00 **(2.9)**	0.94 **(2.9)**
Mar			0.99 **(2.9)**	1.00 **(2.9)**	1.00 **(2.9)**	1.00 **(2.9)**	1.00 **(2.9)**	1.00 **(2.9)**	1.00 **(2.9)**	0.98 **(2.9)**	0.96 **(2.9)**
Apr				0.27 (11.4)	-0.06 (57.1)	0.50 (5.7)	0.27 (14.3)	0.27 (17.1)	0.18 (8.6)	0.33 (5.7)	0.94 **(2.9)**
May					-0.04 (48.6)	0.22 (20)	0.08 (37.1)	0.00 (34.3)	0.12 (20)	0.51 (5.7)	0.96 **(2.9)**
Jun						0.16 (22.9)	0.08 (25.7)	0.12 (25.7)	-0.01 (60)	0.35 (5.7)	0.94 **(2.9)**
Jul							-0.03 (40)	0.19 (11.4)	0.28 (8.6)	0.50 (5.7)	0.94 **(2.9)**
Aug								-0.05 (48.6)	-0.02 (45.7)	0.40 (8.6)	0.96 **(2.9)**
Sep									-0.19 (88.6)	0.52 (**2.9**)	0.93 **(2.9)**
Oct										0.40 (5.7)	0.90 **(2.9)**
Nov											0.80 **(2.9)**

Significant (0.01) correlations are bolded. Numbers in brackets are expressed as percentages (%).

### Abundance and diversity of juvenile fishes in relation to abiotic factors

All of the indices studied for the entire sample (abundance, number of species, richness, diversity and evenness) were positively correlated with tide and temperature, and negatively with salinity, with varying levels of statistical significance (Table 4). The abundance of six dominant species was correlated with the aforementioned abiotic factors to test whether the effect of abiotic factors on the fish community was species-specific. Five of these species exhibited an identical trend as the entire sample, but *Liza carinata* was negatively correlated with tidal level, positively correlated with salinity, and negatively correlated with temperature (Table 4).

**Table 4:** Correlation analysis between fish and abiotic factors in the Maowei Sea.

	T type	T level	Salinity	Temp
**Overall species**				
Abundance	-0.11	0.29	-0.45**	0.43**
No. of species	0.21	0.32*	-0.27	0.13
Species richness (D_ma_)	0.22	0.20	-0.32*	0.30*
Species diversity (H’)	0.07	0.25	-0.26	0.20
Species evenness (J’)	0.05	0.15	-0.29*	0.28
**Abundant species**				
*Pseudogobius javanicus*	0.00	0.29*	-0.25	0.31*
*Omobranchus elegans*	-0.30^*^	0.06	-0.54**	0.44**
*Nemipterus virgatus*	-0.18	0.23	-0.46**	0.31*
*Konosirus punctatus*	-0.13	0.24	-0.45**	0.30*
*Engraulis japonicus*	0.18	0.03	-0.26	0.24
*Liza carinata*	0.05	-0.04	0.33*	-0.38**

T type is tidal rhythm type (diurnal or semi-diurnal), T level is tidal level, and Temp is temperature.

* indicates that the correlation is statistically significant (0.05 level), and

** highly significant (0.01 level). All statistically significant values are bolded. For tide type and level, positive values indicate a positive correlation with diurnal and high tide. ANOVA was used to assess statistical significance.

## Discussion

We studied species composition and abundance of juvenile fish (which includes the larvae as well) in the Maowei mangroves during one calendar year. A total of 11 691 individuals collected belonged to 24 species and 15 families, wherein six perciform species constituted 93% of the total sample. Particularly abundant were *Omobranchus elegans* and *Pseudogobius javanicus*. Abundance was the highest in summer and lowest in winter. Analyses indicate that diversity (highest in May) and richness indices were positively correlated with tide and temperature, and negatively with salinity. Finally, seasonal variations played a more important role in the fish assemblage structure than tidal rhythm, with differences particularly pronounced between colder and warmer months.

### Fish abundance and diversity in the Maowei mangroves

#### Overall abundance and diversity

The number of species (24) recorded in this research was very low in comparison to mangroves worldwide, where species richness is usually larger than 40, and often even larger than 100 [5]. Although latitude does have some impact, species numbers and composition in mangroves are mostly influenced by the size and type of mangrove system, specifically by habitat diversity, structure, and hydrology [5,19]. Additionally, some studies indicate that seagrass beds are the preferred nursery habitat for fish species in the Indo-Pacific, which is in connection with tidal regime and salinity [24]. Thus low species diversity in the Maowei Sea was also probably caused by a large number of factors. These include the absence of nearby coral reefs, resulting in the absence of a large number of fish species associated predominantly with coral reef habitats, which often use mangroves as a nursery [5]. Furthermore, since the late 1980s, the economy of the Beibu Gulf Economic Rim has been experiencing strong growth, resulting in high level of eutrophication and strong negative impacts on the abundance and diversity of benthic organisms in the Maowei Sea [15]. The depletion of this important food source may have produced significantly negative impacts on fish abundance and diversity as well. Low diversity is also probably a reflection of the overall low fish diversity in the entire eastern part of the Beibu Gulf, as evidenced by a 17 month-long study of ichthyoplankton, where only 61 species were identified [25]. In that study, fish eggs were numerically dominated by Carangidae 19.3%, Clupeidae 7.59%, Sciaenidae 7.00%, Engraulidae 6.85%, Leiognathidae 6.52% and Nemipteridae 5.15%, whereas fish larvae were dominated by Engraulidae 33.78%, Gobiidae 8.49%, Bregmacerotidae 7.59% and Leiognathidae 6.21% [25]. In comparison to that study, a notable difference in our findings is the complete absence of Carangidae from the Maowei mangroves. Similarity was higher regarding the fish larvae, as only Leiognathidae were absent from the Maowei mangroves (among these six most abundant families). Thus, although the abundance and species composition of juvenile fish in the mangroves of the Maowei Sea indicate that the mangrove estuary acts as a nursery and feeding ground in the early life history of many fish species, the species composition does not appear to be completely representative of the eastern part of Beibu Gulf [25]. Furthermore, species composition in the Maowei mangroves is very uneven, as only two species, *Pseudogobius javanicus* and *Omobranchus elegans*, constitute over 80% of the total catch, whereas the remaining 20% was shared by the remaining 22 species (see Table 1). This type of fish community structure, completely dominated by several species, is a common feature of estuarine fish assemblages [26,27]. The most abundant family were Gobiidae, representing about 54% of the fish species in the Maowei mangroves. This is rather common, as Gobiidae are typical estuarine residents whose larvae often dominate estuarine habitats (including the mangroves in SE Asia), partly because they are the most speciose family of estuarine fishes and have a relatively long larval phase of approximately 40 days [8,18].

#### Seasonal variability in abundance and species composition

Mangrove habitats are very suitable for fish larvae and juveniles because of the high structural complexity providing shelter, relatively higher maximum water temperatures in shallow stagnant waters (in comparison to the open sea), and diverse and abundant food supply that can meet the needs of a number of different species with very different feeding habits [5,6,13]. However, although some studies indicate that the list of fish species that spawn within estuaries includes at least several families, typically only a few species of so-called permanent residents, such as gobiids, are thought to regularly spawn within estuarine ecosystems [5,18]. Most euryhaline fishes probably enter estuaries as juveniles or postlarvae after spending their larval stage in offshore waters, where adults normally spawn [18]. Among ichthyoplankton in the eastern Beibu Gulf, species diversity was the highest in spring for eggs, and in summer for larvae [25]. We also found high seasonal variability in juvenile fish abundance and species composition, particularly pronounced between summer-autumn (high) and winter-spring (low) seasons. This is also common for many mangrove habitats throughout SE Asia [10]. In spring and winter, when temperatures are low and food less abundant [25], reproductive activity appears to be mostly absent from the Maowei mangrove habitat, and most fish juveniles probably retreat into warmer open waters. Nevertheless, juveniles of some fish species, for example *Pseudogobius javanicus* and *Ophiocara* sp., were present throughout the whole year. Among the species that only appeared seasonally were: *Caragobius urolepis* and *Rhabdosargus sarba* only in spring, and *Ophiocara porocephala* and *Acanthopagrus latus*. Particularly interesting is the appearance of taxonomically unidentified “other”, which were observed only in December and January, but in very large numbers: 189 (48.3% of the total catch) and 115 (69.4%), respectively. It is possible that these were larvae of one or more species that remained in Maowei Mangroves in the following months, when they were large enough to be taxonomically identified. However, as only one specimen was recorded in February, it is more likely that those were larvae of an unidentified species that were present in the Maowei mangroves only during those two months and then retreated back into the open sea as soon as the water temperature started to rise. The presence of juvenile fish in the Maowei mangroves throughout the entire year indicates that despite prominent seasonal differences in abiotic factors the ecosystem provides habitat and/or food sources for fish throughout the entire year.

#### Abiotic factors

Abundance and community structure of fish juveniles in the Maowei estuary were also influenced by abiotic factors. Juvenile fishes were the most abundant, and community complexity the highest, at high tide, when temperature was high and salinity low (Table 2). *Liza carinata* and “other” did not conform to this trend, as they were in negative correlation with tidal level, positive correlation with salinity, and negative correlation with temperature. However, the absence of obvious clustering according to the tidal rhythm (Fig 3) indicates that seasonal variations play a more important role in the fish assemblage structure than tidal rhythm.

Fish species richness and fish abundance in mangrove estuaries are often greater in the wet season than in the dry season, which is probably a reflection of the breeding patterns of fishes and changes in food availability in the estuary [8,18,19]. High rainfall might increase turbidity in estuaries, resulting in decreased predation risk for juvenile fish and associated positive effect on fish abundance [28]. In terms of abundance, Maowei mangroves were no exception, as it began to rapidly decrease with the onset of the dry season in October, and then increase with the onset of the rainy season in April. Correlation with species diversity was less obvious, but the highest values were also recorded during the rainy season, in May and June (Fig 2). Margalef's index of species richness was the lowest in July (the only month when it was lower than Weaver's index of species diversity; Fig 2C). This was probably caused by the fact that several species, such as *Konosirus punctatus*, *Nemipterus virgatus* and *Engraulis japonicus*, reached their maximum abundance in July, leading to a decrease in the proportion of the two species that were dominant throughout the rest of the year, *Pseudogobius javanicus* and *Omobranchus elegans*. These perturbations in the community structure of juvenile fishes were also reflected in a relatively high Pielou's index of evenness value in July (Fig 2C).

It should be noted that the two dominant species, *Pseudogobius javanicus* and *Omobranchus elegans* (together representing 81.68% of the catch), are both demersal species that probably spend their entire life cycles within the estuary [29,30]. Hence, it is rather likely that persistent sampling six to ten times monthly throughout the entire year in the same mangrove channel have resulted in notably reduced numbers of these two species on the sampled locality. This is the most likely explanation for the much lower fish abundance observed in May and June 2013 (35–40) in comparison to the period from July to September 2012 (109–162).

An intriguing feature of the fish assemblage in the Maowei estuary is a strong uptick in species diversity, reflected in Margalef's index of species richness as well, in February, followed by a decrease in March, when the lowest number of individuals and species was recorded. This indicates that a number of species migrate to the Maowei mangroves in February, which might be in correlation with the sudden increase in water temperature observed during this period (Fig 2A).

Apart from being passively transported by sea currents [18,25,26], fish larvae also possess the swimming ability that enables them to actively choose habitats [31–34]. Salinity and temperature are considered to be the most important abiotic factors to fish larvae and juveniles [18]. Prominent seasonal variations in salinity in mangrove estuaries are common [19], but our results are in contrast with the positive correlation between salinity and fish abundance observed in the Tanshui River in Taiwan [26]. However, in that study, correlation coefficients varied among species. As there is no overlap between the dominant species in the Maowei and Tanshui estuaries, it is highly likely that this discrepancy is a reflection of interspecific life history differences. In addition, it has been proposed that, along with passive transport by tidal currents, larvae and juveniles actively migrate and select preferred habitats in the Tanshui estuary [26]. It was also previously observed that all larval stages of the Gobiidae were more abundant in the less saline, zooplankton-richer, waters inside the mangroves [18]. As salinity in the Maowei mangroves was lower than in some comparable Asian mangroves [8,18], and as Gobiidae were the dominant family in our research, this explains the observed negative correlation between salinity and fish abundance.

### Importance for fisheries and biodiversity

As a result of somewhat slower pace of destruction in comparison to other localities in China, the relative importance of mangroves in Guangxi province has been constantly growing (on the national level) during the last 25 years [15]. With over 520 commercially exploited fish species, Beibu Gulf is among the areas with highest fishery resource productivity in China, with fisheries being a major industry in the region [15,25]. The increasing fishing pressure during the past 40 years was accompanied by drastic resource depletion and a major shift in the ecosystem structure, with the biomass of long-lived, high trophic level, piscivorous fishes declining by 94% between 1960 and 1999, while short-lived, small fishes and benthic invertebrates gradually became dominant [21,22]. Thus, conservation of Beibu mangroves would be of particular importance (both from the commercial and conservation perspective) if it can be shown that they support resident fish communities that are functionally important as an intermediate trophic level for piscivorous fish [19]. Although our results provide an indication that this may be true, further studies are needed to strengthen this link. Seasonal changes in taxonomic composition, abundance and distribution of fish larvae and juveniles are an important factor for fishery resources research and management [25]. Larval recruitment and survival in the mangroves will have a strong bearing on structure and abundance of the fish community in nearby coastal waters, and likely on the local fishing industry [5,12,13,18]. Among the species found in this research (see Table 1), of importance for commercial fisheries are: Cynoglossidae, Tetraodontidae, all three Sparidae species (highly commercial), *Sardinella* (highly commercial), *Konosirus punctatus* (2.24% in this study, minor commercial value), *Liza carinata* (1.2%, commercial), *Engraulis japonicus* (1.44%, highly commercial), and finally *Nemipterus virgatus* (6.3%), which is one of the most important species of commercial fisheries in the northern South China Sea, and listed in the IUCN as vulnerable [30,35–37]. Thus, although the extent of the direct significance of mangroves for the large-scale commercial fisheries is still debated [5,12,13], this research presents a strong evidence that Maowei mangroves are a nursery for at least some commercially important fishes in the Beibu Gulf. Furthermore, regardless of the extent of the direct economic value of Maowei mangroves for the large-scale commercial fisheries in the Beibu Gulf, the four families found in this research (Blenniidae, Ambassidae, Eleotridae and Syngnathidae) that were completely absent from the ichthyoplankton throughout the rest of the eastern Beibu Gulf [25] evidence the indispensability of this biotope for fish diversity in the Beibu Gulf. Finally, the presence of species that have been recognised as vulnerable further emphasises the importance of this biotope for the conservation of fish species that are either overfished or in any other way endangered by natural and anthropogenic factors.

## Materials and methods

### Study area

Maowei Sea (Guangxi Province) is a large estuary located at the far northern end of the Beibu Gulf, South China Sea (Fig 1A). It is formed by several rivers of varying sizes (Qing and Maoling being the largest), and separated from the Beibu Gulf by a relatively narrow (min ≈ 200 m) channel [38]. Even though the surface area is rather large (approximately 135 km^2^), depth is quite shallow: 0.1–5 m [14]. The most common mangrove species are *Bruguiera* *gymnorrhiza*, *Kandelia candel* and *Rhizophora stylosa*. The climate is monsoon subtropical with long, hot and rainy summers and short, dry, mostly frost-free winters [39]. The average annual temperature is 22°C, rainfall is about 2000 mm, and wind speed is 2.6 m/s [17]. The region has irregular diurnal tides, with maximum and minimum tidal range of 4.04 and 0.05 m, respectively, and the mean variation of 2.52 m [38].

### Sampling procedure

Fish larvae and juveniles were sampled from July 2012 through June 2013 among mangrove islands within the channel that separates the Maowei Sea from the Beibu Gulf (see Fig 1B for geographic coordinates). The channel where the sampling was conducted is approximately 50 m wide at its narrowest point and ≈ 0.8–1 m deep during an average low tide. During the high tide, most of the mangroves and islands become inundated and the channel becomes much wider and deeper (see previous section and Fig 1B). Fish were sampled using shallow-water plankton net: 1.45 m length, 0.5 m diameter, 0.5 mm mesh size. The net was drawn by motorboat at 1.5 to 2 knots near the surface approximately for an hour to make a round trip of 2.5 km (2 x 1250 m): from start point to end point, and then back to start point (Fig 1B). The net was emptied approximately once every 20 minutes. Environmental factors, water temperature and salinity (CT-3080 salinometer), were measured *in situ* during every pause for net-emptying. Sampling was undertaken on 103 days, twice on each sampling day (206 samples in total): once during high tide and once during low tide (both neap and spring). In April, May, July, August and September, sampling was undertaken on ten different days (5×20 samples); in October, November and December on nine days (3×18 samples); in June on eight days (16 samples); and in January, February and March on six days (3×12 samples). The number of sampling trips was reduced in winter/early spring months as juvenile fish (and larvae) abundance decreased strongly during this period, and we feared that often sampling might depopulate the locality. Adult fish, identified by the presence of scales [40], were removed from the samples (returned to water), whereas larvae and juveniles were immediately euthanized in MS-222 (Sigma-Aldrich, USA) at 250 mg/L concentration and fixed in 10% buffered formalin-seawater solution. The handling of animals was conducted in accordance with the guidelines for the care and use of animals for scientific purposes set by the Institutional Animal Care and Use Committee of the Guilin University of Technology, Guangxi, China and the EU Directive 2010/63/EU for animal experiments. The permit to conduct this study was obtained by the Guangxi University, and issued by the GuangXi Laboratory Animal Public Service Center (permit number: SCXK Guangxi 2013–0003). As the sampled location is not a part of the protected area, no special permits were required for the sampling.

### Data analysis

The sampled juveniles and larvae were identified to the lowest possible taxonomic level under different microscopes (light and inverted) and different magnifications (10–40×), according to procedures and keys outlined in a number of publications [41–45]. For the purpose of this analysis, juveniles and larvae were analysed together; thus the term 'juveniles' here refers to both life stages. Cumulative monthly samples were pooled and species richness and abundance expressed as the mean number of species and individuals per 2.5 km tow, respectively. Community structure was evaluated using Shannon-Weaver's index of species diversity (H’) [46], Margalef’s index of species richness (D_ma_) [47] and Pielou's index of evenness (J’) [48]. Cluster analysis based on Bray-Curtis similarity measure was used to examine the impact of season (month) and tide on fish community assemblage [49]. Prior to the analyses, abundance data were log(x+1) transformed in order to normalise distributions and stabilise variances. Analysis of similarity (ANOSIM) was used to determine whether fish assemblages differed significantly between months. The relationship (correlation) between abundance and abiotic factors was assessed for six most abundant species. All analyses were performed using Primer-E5 [49] and Statistica 19.0 (StatSoft Inc., Tulsa, USA) programs.
